# Supplementary material for: Discoveries of Exoribonuclease-Resistant Structures of Insect-Specific Flaviviruses Isolated in Zambia
Source: Viruses. 2020 Sep 11;12(9):1017. doi: 10.3390/v12091017 (PMC7551683; doi:10.3390/v12091017)
Supplement: Supplementary file 1 [file viruses-12-01017-s001.pdf]

viruses

Supplementary

Discoveries of Exoribonuclease-Resistant Structures of Insect-Specific Flaviviruses Isolated in Zambia

Christida E. Wastika [1], Hayato Harima [2], Michihito Sasaki [1], Bernard M. Hang'ombe [3], Yuki Eshita [2], Yongjin Qiu [2], William W. Hall [4,5,6], Michael T. Wolfinger [7,8,*], Hirofumi Sawa [1,5,6] and Yasuko Orba [1,5,*]

[1] Division of Molecular Pathobiology, Research Center for Zoonosis Control, Hokkaido University, Sapporo 001-0020, Japan; christida@czc.hokudai.ac.jp (C.E.W.); m-sasaki@czc.hokudai.ac.jp; (M.S.); h-sawa@czc.hokudai.ac.jp (H.S.)

[2] Hokudai Center for Zoonosis Control in Zambia, Research Center for Zoonosis Control, Hokkaido University, Lusaka 10101, Zambia; harima@czc.hokudai.ac.jp (H.H.); yeshita@czc.hokudai.ac.jp (Y.E.); yongjin_qiu@czc.hokudai.ac.jp (Y.Q.)

[3] Department of Para-Clinical Studies, School of Veterinary Medicine, University of Zambia, Lusaka 10101, Zambia; mudenda68@yahoo.com

[4] Center for Research in Infectious Disease, University College Dublin, Dublin, Ireland; william.hall@ucd.ie

[5] International Collaboration Unit, Research Center for Zoonosis Control, Hokkaido University, Sapporo 001-0020, Japan

[6] Global Virus Network, Baltimore, MD 21201, USA

[7] Department of Theoretical Chemistry, University of Vienna, 1090 Vienna, Austria

[8] Research Group Bioinformatics and Computational Biology, Faculty of Computer Science, University of Vienna, 1090 Vienna, Austria

* Correspondence: michael.wolfinger@univie.ac.at (M.T.W.); orbay@czc.hokudai.ac.jp (Y.O.)

Abstract: To monitor the arthropod-borne virus transmission in mosquitoes, we have attempted both to detect and isolate viruses from 3304 wild-caught female mosquitoes in the Livingstone (Southern Province) and Mongu (Western Province) regions in Zambia in 2017. A pan-flavivirus RT-PCR assay was performed to identify flavivirus genomes in total RNA extracted from mosquito lysates, followed by virus isolation and full genome sequence analysis using next-generation sequencing and rapid amplification of cDNA ends. We isolated a newly identified Barkedji virus (BJV Zambia) (10,899 nt) and a novel flavivirus, tentatively termed Barkedji-like virus (BJLV) (10,885 nt) from *Culex* spp. mosquitoes which shared 96% and 75% nucleotide identity with BJV which has been isolated in Israel, respectively. These viruses could replicate in C6/36 cells but not in mammalian and avian cell lines. In parallel, a comparative genomics screening was conducted to study evolutionary traits of the 5′- and 3′-untranslated regions (UTRs) of isolated viruses. Bioinformatic analyses of the secondary structures in the UTRs of both viruses revealed that the 5′-UTRs exhibit canonical stem-loop structures, while the 3′-UTRs contain structural homologs to exoribonuclease-resistant RNAs (xrRNAs), SL-III, dumbbell, and terminal stem-loop (3′SL) structures. The function of predicted xrRNA structures to stop RNA degradation by Xrn1 exoribonuclease was further proved by the in vitro Xrn1 resistance assay.

Keywords: Barkedji virus; Barkedji-like virus; dISFVs; 5′-UTR; 3′-UTR; RNA secondary structure

Supplementary Table S1. Nucleotide and amino acid identity comparison between Barkedji virus Zambia, Barkedji-like virus and other flaviviruses.

Ref. number	Virus	5′-UTR (nt)	AnchC-C		prM		M protein		Envelope		NS1 protein	
			aa	ID (%)	aa	ID (%)	aa	ID (%)	aa	ID (%)	aa	ID (%)
**LC497470**	**Barkedji virus strain Zambia**	**102**	125	-	92	-	75	-	503	-	350	-
LC497469	Barkedji-like virus	103	127	86.6	92	91.3	75	88.0	503	90.4	350	92.2
AUS91146.1	Barkedji virus strain Oman	44*	125	100.0	92	100.0	75	100.0	503	100.0	350	100.0
YP_009056848.1	Lammi virus**	-	122	25.4	93	47.1	76	46.0	499	43.9	353	49.2
ARO84721.1	Dengue virus 1 TM 248	160	114	37.0	91	43.3	75	39.7	495	43.8	352	47.2
AIU47320.1	Dengue virus 2 New Guinea C	96	114	34.3	91	42.2	75	41.0	495	42.4	352	46.8
ADC35596.1	Dengue virus 3 GZ1D3	94	114	37.2	91	43.0	75	42.4	493	42.6	352	46.8
AKQ00028.1	Dengue virus 4 BR/SJRP/500/2012	112	113	37.0	91	41.1	75	42.6	495	44.6	352	48.5
YP_002790881.1	Zika virus MR766	106	122	37.6	93	39.0	75	41.3	500	43.8	352	53.6
YP_004734464.1	Tembusu virus	94	120	35.9	92	38.8	75	42.6	501	45.6	352	50.2
AAS59402.1	Usutu virus strain Vienna 2001	96	126	29.9	92	44.4	75	52.0	500	48.1	352	50.2
NP_059434.1	Japanese Encephalitis virus	95	127	31.0	92	40.0	75	48.0	500	46.4	352	51.4
AIW82672.1	West Nile virus BD-AUT	96	123	33.6	92	46.1	75	46.6	501	46.2	352	51.7
AIZ07889.1	Yellow Fever virus strain 8A-2014**	-	121	29.1	89	38.0	75	40.0	493	39.5	352	48.1
NP_619758.1	Modoc virus	109	110	20.6	87	33.8	75	18.7	482	32.4	353	39.2
NP_043135.1	Tick-borne encephalitis virus	132	112	19.0	93	40.0	75	33.3	496	36.0	352	39.4
NP_620099.1	Powassan virus	111	110	28.2	93	40.7	75	28.0	497	36.2	353	38.7
YP_899469.2	Culex flavivirus	91	138	19.6	84	26.3	60	46.1	427	17.3	393	28.7
NP_891560.1	Kamiti River virus	96	143	30.7	81	34.4	62	18.5	432	19.8	390	27.9

Ref. number	Virus	NS2A protein		NS2B protein		NS3 protein		NS4A protein		2K		NS4B	
		aa	ID (%)	aa	ID (%)	aa	ID (%)	aa	ID (%)	aa	ID (%)	aa	ID (%)
LC497470	Barkedji virus strain Zambia	233	-	129	-	621	-	126	-	23	-	255	-
LC497469	Barkedji-like virus	233	81.5	129	90.6	621	92.4	126	84.9	23	100.0	255	91.7
AUS91146.1	Barkedji virus strain Oman	233	99.1	129	100.0	621	99.5	126	99.2	23	100.0	255	99.2
YP_009056848.1	Lammi virus**	227	37.5	131	29.2	621	54.8	126	35.6	23	45.0	255	36.0
ARO84721.1	Dengue virus 1 TM 248	218	28.0	130	28.0	619	54.0	127	38.5	23	45.0	249	38.5
AIU47320.1	Dengue virus 2 New Guinea C	218	25.9	130	34.9	618	54.5	127	38.2	23	35.0	248	38.5
ADC35596.1	Dengue virus 3 GZ1D3	218	25.7	130	35.3	619	55.3	127	37.3	23	45.0	248	39.3

AKQ00028.1	Dengue virus 4 BR/SJRP/500/2012	218	25.9	130	29.8	618	54.1	127	36.5	23	21.4	245	39.1
YP_002790881.1	Zika virus MR766	226	40.3	130	30.9	617	55.3	127	40.8	23	40.0	251	41.3
YP_004734464.1	Tembusu virus	227	36.5	131	25.0	619	56.4	126	43.6	23	39.1	254	44.1
AAS59402.1	Usutu virus strain Vienna 2001	227	36.5	131	32.9	619	54.9	126	40.8	23	39.1	258	38.7
NP_059434.1	Japanese Encephalitis virus	227	36.3	131	27.1	619	56.5	126	42.6	23	39.1	255	37.8
AIW82672.1	West Nile virus BD-AUT	231	35.4	131	28.4	619	54.9	122	39.6	27	47.8	256	37.5
AIZ07889.1	Yellow Fever virus strain 8A-2014**	224	28.9	130	22.9	623	50.9	126	30.9	23	40.0	250	34.7
NP_619758.1	Modoc virus	221	17.1	132	29.7	618	46.1	121	27.8	23	29.4	254	22.2
NP_043135.1	Tick-borne encephalitis virus	230	23.3	131	27.2	621	45.0	126	31.4	23	30.7	252	26.1
NP_620099.1	Powassan virus	230	22.6	131	24.1	622	42.9	126	32.5	23	22.2	252	27.7
YP_899469.2	Culex flavivirus	202	16.9	146	13.1	588	34.2	156	31.4	23	26.3	257	28.2
NP_891560.1	Kamiti River virus	207	16.6	149	22.9	587	32.8	135	19.1	23	45.4	260	54.5

**Ref. number**	**Virus**	**NS5**		**3′-UTR (nt)**	**TOTAL**	
		**aa**	**ID (%)**		**aa**	**ID (%)**
LC497470	Barkedji virus train Zambia	905	-	483	3,437	-
LC497469	Barkedji-like virus	905	92.1	462	3,439	90.6
AUS91146.1	Barkedji virus strain Oman	905	99.3	338*	3,437	99.5
YP_009056848.1	Lammi virus**	908	61.9	-	3,434	48.4
ARO84721.1	Dengue virus 1 TM 248	899	62.1	404	3,392	47.3
AIU47320.1	Dengue virus 2 New Guinea C	900	62.5	454	3,391	47.1
ADC35596.1	Dengue virus 3 GZ1D3	900	61.8	443	3,390	47.5
AKQ00028.1	Dengue virus 4 BR/SJRP/500/2012	900	61.7	152	3,387	47.7
YP_002790881.1	Zika virus MR766	903	63.0	431	3,419	50.0
YP_004734464.1	Tembusu virus	905	62.2	621	3,425	49.9
AAS59402.1	Usutu virus strain Vienna 2001	905	64.1	668	3,434	50.1
NP_059434.1	Japanese Encephalitis virus	905	63.4	585	3,432	49.8
AIW82672.1	West Nile virus BD-AUT	905	62.2	630	3,434	49.6
AIZ07889.1	Yellow Fever virus strain 8A-2014**	905	59.1	-	3,411	45.0
NP_619758.1	Modoc virus	898	52.3	369	3,374	37.4
NP_043135.1	Tick-borne encephalitis virus	903	57.7	767	3,414	40.5
NP_620099.1	Powassan virus	903	58.0	483	3,415	40.2
YP_899469.2	Culex flavivirus	889	42.2	657	3,363	28.4
NP_891560.1	Kamiti River virus	888	44.2	1,208	3,357	28.3

*sequence was not completed; **sequence only including open reading frame.

Supplementary Table S2. List of primer sets used in this study.

Primer name	Sequence 5′→ 3′
	**Pan-flavivirus primer set**
Flavi all S	TACAACATGATGGGGAARAGAGARAA
DEN4 F	TACAACATGATGGGRAAACGTGAGAA
Flavi all AS 2	GTGTCCCAGCCNGCKGTGTCATCWGC
	**5′-RACE primer set**
BJVrace 5P-964R	P- CTCCATTTCCRTGA
BJV40race 820 F1	TGACACTAATGATCGTGATGA
BJV31race 825 F1	GACATCGACACCAATGAGC
BJV40race 887 F2	ATGGACGTTGTCAAACTGGA
BJV31race 883 F2	CACTATGGGAGATGTCAAGG
BJV40race 566 R1	TTCAAATCCTTGAGCATGTC
BJV31race 578 R1	CGCTTCCTGGAATTCAGATC
BJV40race 523 R2	CCAAAACTTTGCTACCTTCC
BJV31race 523 R2	CCAGAACCTTACTTCCTTCC
	**3′-RACE primer set**
BJV40race 10270 F	ACATGGTCACTGCACGGAAA
BJV31race 10270 F	CACTTGGTCACCTGCATGGAAA
	**vRNA qRT-PCR primer set**
BJVzall 2622F	GAAGCACTGGGGAAGGAGAC
BJV L31 2622F	GAAGCATTGGGGCAGGAGAC
BJVzall 2807R	TCATCATTGTCCTTCTCATTCA
	**In vitro Xrn1 resistance assay primer set**
BJV L31 T7-10450F	ATGTAATACGACTCACTATAGGGAAAAAGAAATCAGAATAAGG
BJLV M40 T7-10422F	ATGTAATACGACTCACTATAGGGAAATTTGAAAGAAAGTGGGA
BJLV M40 T7-10422F dHP10452-10467	ATGTAATACGACTCACTATAGGGAAATTTGAAAGAAAGTGGGAAAAAGAAAAAACGACACGAGT
BJLV M40 10558R	TAAGCGGCCCAACAATTTCC
BJV L31 10570R	TTAGCGGTCCAACTCAATCC
**Xrn1 stop point identification primer**	
BJV/BJLV xrRNA sequence	FAM-AGAGGUGGCAAGCAUAAGCC
